# Polycation–Polyanion Architecture of the Intermetallic Compound Mg_3−*x*_Ga_1+*x*_Ir

**DOI:** 10.3390/molecules27030659

**Published:** 2022-01-20

**Authors:** Olga Sichevych, Yurii Prots, Walter Schnelle, Frank R. Wagner, Yuri Grin

**Affiliations:** Max-Planck-Institut für Chemische Physik Fester Stoffe, Nöthnitzer Str. 40, 01187 Dresden, Germany; olga.sichevych@cpfs.mpg.de (O.S.); prots@cpfs.mpg.de (Y.P.); walter.schnelle@cpfs.mpg.de (W.S.); Frank.Wagner@cpfs.mpg.de (F.R.W.)

**Keywords:** intermetallic compound, chemical bonding, electron localizability, QTAIM, 8–*N* rule, polyanion, polycation

## Abstract

Mg_3−*x*_Ga_1+*x*_Ir (*x* = 0.05) was synthesized by direct reaction of the elements in welded tantalum containers at 1200 °C and subsequent annealing at 500 °C for 30 days. Its crystal structure represents a new prototype and was determined by single-crystal technique as follows: space group *P*6_3_/*mcm*, Pearson symbol *hP*90, *Z* = 18, *a* = 14.4970(3) Å, *c* = 8.8638(3) Å. The composition and atomic arrangement in Mg_3_GaIr do not follow the 8–*N* rule due to the lack of valence electrons. Based on chemical bonding analysis in positional space, it was shown that the title compound has a polycationic–polyanionic organization. In comparison with other known intermetallic substances with this kind of bonding pattern, both the polyanion and the polyanion are remarkably complex. Mg_3−*x*_Ga_1+*x*_Ir is an example of how the general organization of intermetallic substances (e.g., formation of polyanions and polycations) can be understood by extending the principles of 8–*N* compounds to electron-deficient materials with multi-atomic bonding.

## 1. Introduction

During their scientific history, intermetallic compounds have challenged chemists because their compositions and crystal structures cannot be rationalized or understood using usual valence rules. Recognizing this fact very early in the 20th century, Linus Pauling, in his lecture on the occasion of the receipt of the Theodore William Richards Medal of the ACS on 8 May 1947, stated that, “One great problem in structural chemistry which still awaits satisfactory solution is that of the structure of metals and intermetallic compounds” [1].

From the point of view of the periodic table of elements, intermetallic compounds belong to the inorganic materials but contain only elements located on the Zintl line and to the left of it. The main difference between the “classical” inorganic and intermetallic compounds is the low number of electrons in the last shell per atom (ELSA) in the latter [2]. This hinders the application of bonding concepts based on the 8–*N* rule to understand the composition and structure of intermetallic compounds. Instead, it was suggested to extend the 8–*N* concept by taking into account the participation of *d* orbitals (penultimate shell) in bonding events (18–*N* rule). Furthermore, this situation involves the concept of multi-atomic bonding. 

The analysis of chemical bonding in position space has developed in the last decades into an important quantum–chemical tool for resolving the stoichiometric and structural organization of intermetallic materials. Using this technique and under ELSA deficiency conditions, it was shown that the formation of polyanions can be observed in Zintl phases (e.g., [3,4,5]) and in compounds with lower ELSA and multi-atomic bonding (e.g., [6]). Moreover, the “excess” electrons not consumed in the anionic substructure can be used for the formation of polycations, such as in LuGe with Zintl-like electron counting [7] or in Sr_3_Li_5_Ga_5_, representing a polycation requiring less electrons for its stabilization compared with a Zintl count [8]. While the polyanionic entities in these materials are either infinite (chain-like ^1^_∞_[Ge]) or island-like (bell-like [Ga_5_]), the observed size of the polycations is limited to either four-atom tetrahedral [Lu_4_] or six-atom octahedral [Sr_6_] entities. 

The idea to search for polyanionic–polycationic systems among the compounds of magnesium with gallium and iridium originates from a known series of new ternary magnesium transition metal–boron compounds [9,10,11], most of which represent new prototypes with interesting structural peculiarities. As an example, the structure of Mg_1−*x*_BRh is highlighted by the 2D polyanion ^2^_∞_[BRh] formed by three-bonded boron and two-bonded rhodium atoms [12]. On the other hand, iridium-containing intermetallic compounds are suggested as catalysts or precursors for catalysts in oxygen evolution reactions during water electrolysis (e.g., Hf_2_B_5_Ir_2_ [13]). Thus, we extend the investigation to the Mg–Ga–Rh and Mg–Ga–Ir systems. As the first result of these experiments, the new compound Mg_3−*x*_Ga_1+*x*_Ir was obtained. This represents the first system among intermetallic compounds with extended two-dimensional (2D) polyanions ^2^_∞_[Ga_3_Ir_2_] interpenetrated by one-dimensional polycations ^1^_∞_[IrMg_6_Mg_6/2_]. 

## 2. Experimental and Computational Details

Several samples based on the composition of Mg_3_GaIr were prepared from high-purity elements: Mg, chips, 99.99%; Ir, foil, 99.99%; Ga, pellets, 99.999%. The mixture of components was loaded into Ta tubes under an argon pressure of about 800 mbar. The sealed tantalum tubes were subsequently enclosed into evacuated silica ampoules to prevent oxidation of tantalum during the high-temperature treatment. The mixture was heated at 1200 °C for 2 h. After cooling down to 500 °C at a rate of 50 K/h, the sample was thermally treated for 30 days and finally quenched in cold water. The obtained product was well crystallized and displayed metallic luster. 

The samples were characterized by X-ray powder diffraction using an imaging plate Guinier Camera (HUBER G670 Co*K_α_*_1_ radiation, *λ* = 1.788965 Å, 8 × 15 min scans). Unit cell parameters were refined by a least squares procedure using the positions of 47 peaks (14° ≤ 2*θ* ≤ 100°) extracted from a powder X-ray diffraction pattern measured with LaB_6_ as internal standard (*a* = 4.15692 Å). Indexing of the diffraction peaks was controlled by intensity calculations using the positional parameters from the refined structure. 

The crystal structure of Mg_3−*x*_Ga_1+*x*_Ir was investigated using single-crystal diffraction data. The data collection was performed on a RIGAKU AFC-7 diffractometer equipped with a Mercury CCD detector. The measured intensities were corrected for absorption using a multi-scan routine [14]. All relevant information concerning the data collection and handling are summarized in Table 1. Atomic parameters were standardized by the STRUCTURE TIDY program [15,16]. All other crystallographic calculations were performed with the WinCSD program package [17]. Interatomic distances were calculated using lattice parameters obtained from Guinier powder X-ray diffraction data.

Microstructure analysis was performed on the annealed samples using optical light microscopy (Zeiss Axioplan 2) as well as scanning electron microscopy (SEM, Philips XL 30). The polished ingots were prepared under inert conditions in an argon-filled glovebox. Silicon carbide (SiC) paper was used for grinding, and diamond polishing with at least ¼ µm diamond powder was applied for finishing. The microstructure surface was cleaned in a hexane bath from the paraffin oil, which acts as lubricant for the preparation of microstructures. 

The standard-less energy-dispersive X-ray absorption analysis (EDXS) was performed on an EDAX system attached to the SEM with an LaB_6_ cathode. The spectra, recorded at 25 kV by the Si(Li) drift detector, solely showed the presence of the constituent elements of the title ternary compound.

Magnetic susceptibility was measured at external fields *µ*_0_*H* between 2 mT and 7 T in the temperature range of 1.9 K–400 K in a SQUID magnetometer (MPMS XL-7, Quantum Design). Electrical resistivity was determined using a four-probe *dc* method between 4 K and 320 K.

The TB-LMTO-ASA program package [18,19] with exchange correlation potential (LDA) after Barth and Hedin [20] and the all-electron, local-orbital, full-potential method (FPLO) within the local density approximation [21], while the Perdew–Wang parametrization [22] was employed for quantum chemical calculations on an ordered model with Mg_3_GaIr. Experimentally obtained lattice parameters and atomic coordinates were employed for the calculations. The analysis of the chemical bonding in the position space [23,24] was performed using the electron localizability approach. For this purpose, the electron localizability indicator (ELI) in its ELI-D representation [25,26] and the electron density (ED) were calculated with specialized modules using LMTO-ASA [18,19] and the FPLO codes [27]. The topologies of ELI-D and ED were evaluated by means of the DGrid program [28]. The atomic charges from ED and bond populations for bonding basins from ELI-D were obtained via the integration of ED within the basins (space regions), bounded by zero-flux surfaces in the according gradient field. This procedure follows the Quantum Theory of Atoms in Molecules (QTAIM) [29]. 

## 3. Results and Discussion

### 3.1. Crystal Structure Determination

Analysis of the symmetry equivalent reflections (*R*(int) = 0.061 for *Laue* symmetry 6/*mmm*) and the systematic extinction conditions (*l* = 2*n* + 1 for *h*$\bar{h}$0*l* and 000*l* reflections) clearly indicated *P*$\bar{6}$*c*2, *P*6_3_*mc* and *P*6_3_/*mcm* as possible space groups, of which the centrosymmetric space group *P*6_3_/*mcm* (No. 193) was found to be correct during the structure refinement. The starting model (all iridium, all gallium and some of the magnesium atomic positions) was obtained from direct methods. The remaining magnesium atoms were located from the Fourier difference map. The refinement of the structure model with 4 Mg, 4 Ir and 2 Ga crystallographic positions converged rapidly to an acceptable *R*(*F*) value of 0.028. Nevertheless, a careful inspection of the isotropic displacement parameters showed that the *U*(iso) values for the Mg3 and Mg4 positions (0.0071 Å^2^ and 0.0065 Å^2^, respectively) were markedly smaller than those for the Mg1 and Mg2 sites (0.0129 Å^2^ and 0.0143 Å^2^, respectively). Refinement of the occupancies of the magnesium positions resulted in values of 0.99(1), 1.00(2), 1.05(2) and 1.13(3) for Mg1, Mg2, Mg3 and Mg4, respectively, indicating the presence of stronger scatterers in the Mg3 and Mg4 positions. Subsequently, the structure was refined with a minor admixture of Ga in these sites. This negligibly reduced the reliability factor *R*(F) to 0.027 and simultaneously increased the *U*(iso) of Mg3 and Mg4 positions to 0.0106 Å^2^ and 0.0117 Å^2^, respectively, i.e., toward the values for Mg1 and Mg2 (Table 2). The statistical distribution of Mg and Ga on a crystallographic site is not a rarity, and was previously observed in certain intermetallic phases, e.g., in EuMg*_x_*Ga_4−*x*_ [30]. The crystal structure was finally refined with anisotropic displacement parameters for all positions. The final atomic coordinates and isotropic displacement parameters are listed in Table 2, while the interatomic distances are listed in Table 3; the anisotropic displacements parameters can be obtained from the database (CSD deposition No. 2129909) or from the corresponding author.

### 3.2. Crystal Structure Description 

**Figure 1:**
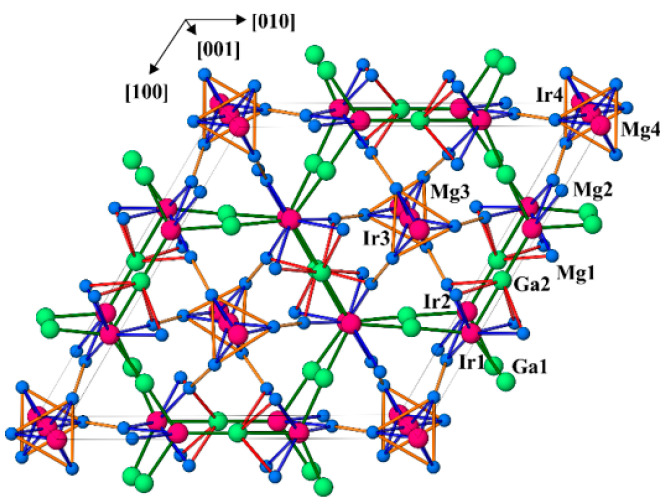
Crystal structure of Mg_3−*x*_Ga_1+*x*_Ir (Mg—blue, Ga—green, Ir—pink) in a projection along [001] with the shortest distances for Ga–Ir (green, <2.6 Å), Mg–Ir (blue, <2.9 Å), Mg–Mg (orange, <2.9 Å) and Mg–Ga (red, <2.9 Å).

The crystal structure of Mg_3−*x*_Ga_1+*x*_Ir represents a new prototype (Figure 1). The sequence of interatomic distances in the environment of the atoms is characteristic for intermetallic compounds (Table 3). The shortest distances are homogeneously distributed within the unit cell (Figure 1). Therefore, two different Ir–Mg distances occur for Ir3 and Ir4 at approximately 2.75 Å and 3.05 Å, respectively. The first value is only slightly larger than the sum of the covalent radii of *r*(Ir) + *r*(Mg) = 2.62 Å [31], while the second one is essentially closer to the sum of the corresponding atomic radii of *r*(Ir) + *r*(Mg) = 2.96 Å [32]. It is notable that comparable Ir–Mg distances of 2.778(2) Å and 2.821(3) Å are also observed for the Ir1 and Ir2 atoms, respectively. The Mg–Mg distances vary between 2.812(3) Å and 3.358(4) Å. For comparison, an average interatomic distance in elemental Mg is 3.201 Å [33]. The Ga–Ir contacts range between 2.398(1) Å and 2.456(1) Å. Comparing these values (*D*(*n*)) with the sum of the single-bond (covalent) radii of *D*(1) = *r*(Ir) + *r*(Ga) = 2.488 Å and by using the equation *D*(*n*) = *D*(1) − 0.71log(*n*) [32], one could estimate the Pauling bond order of *n* = 1.1–1.3 for these Ga–Ir interactions. Short homonuclear contacts for Ir and Ga atoms are not present in the structure of Mg_3−*x*_Ga_1+*x*_Ir. The shortest distances Ir–Ir and Ga–Ga occur at 4.4319(1) Å and 4.036(2) Å, respectively. These values are rather large in comparison to the interatomic distance of 2.715 Å in cubic closest-packed Ir and the average distance of 2.703 Å in the *α*-modification of elemental Ga [33]. Considering only the shortest distances in the crystal structure, a three-dimensional framework including all atoms may be built up (Figure 1). Importantly, this network involves contacts with obviously varying electronegativity differences; according to the Pauling scale, ΔEN(Ir–Mg) = 0.89, ΔEN(Ga–Mg) = 0.50, ΔEN(Ir–Ga) = 0.39. This may hinder the interpretation of the atomic arrangement in this material in terms of the Pauling’s formation of aggregates, having sufficient stability to make them convenient for the chemists to consider them as independent species [32].

In contrast, considering the atomic environments, the coordination polyhedra of iridium (established from the distribution of interatomic distances, cf. Table 3)—the icosahedra Ir3@Mg_12_ and Ir4@Mg_12_—immediately attract attention due to the high symmetry and locations on the three- and six-fold axes. The Ir@Mg_12_ icosahedra are condensed through two opposite faces into infinite columns along the [001] direction. The coordination numbers of the other Ir atoms are smaller (10 for Ir1 and 11 for Ir2) and their polyhedra are less symmetrical. Within the Ir polyhedra, the distances are split into two groups—short Ga–Ir contacts (×3 for each iridium) comparable with the sum of covalent radii and longer Mg–Ir contacts comparable with the metallic radii (cf. above). Such behavior is characteristic for many intermetallic compounds and has its origin in the atomic interactions in this class of materials. 

### 3.3. Band Structure and Properties

**Figure 2:**
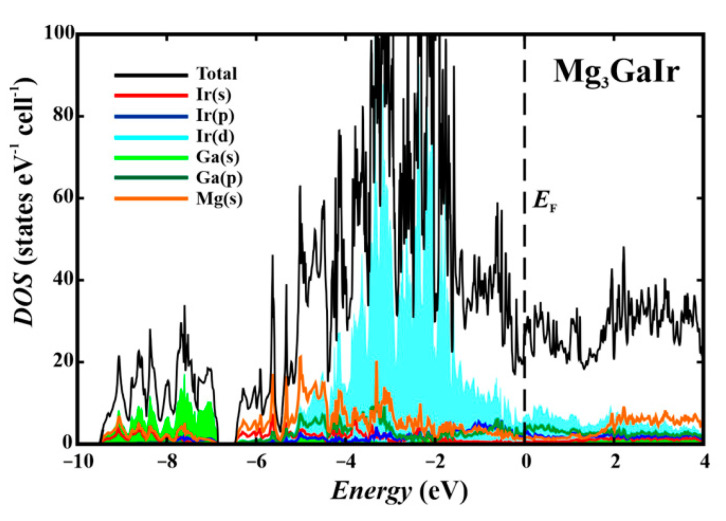
Total electronic density of states (black) and their atomic contributions for Mg_3_GaIr.

The calculated electronic density of states for Mg_3_GaIr has two large regions separated by a gap at around −7 eV (Figure 2). At the Fermi level, only a shallow dip is observed. The low-energy part is formed mainly by the Ga-s states with an admixture of Ir-*s* and Mg-*s*, while the large region between −7 eV and the Fermi level is governed by the *d* states of Ir and *s* and *p* states of Mg, with contributions of the *p* states of Ga. The rather unusually large Mg(*s*) and Mg(*p*) contributions in the same region as Ir(d) indicate a particular Mg–Ir interaction in Mg_3_GaIr. Rather unusual for a typical intermetallic compound is the appearance of a dip in the vicinity of the Fermi level. Similar features were recently observed in Be_22_Pt_5_ [6] and Be_5_Pt [34] and interpreted as a result of complete filling of the valence electron band (in the multi-atomic bond system) supported by a strong charge transfer (cf. below). 

The magnetic susceptibility *χ* = *M*/*H* of Mg_3−*x*_Ga_1+*x*_Ir is small and only weakly temperature-dependent (Figure S1). After considering a small upturn of *χ*(*T*) at the lowest temperatures due to minor paramagnetic impurities, a constant diamagnetic susceptibility *χ*_0_ of −36(10) × 10^−6^ emu mol^−1^ at *T* = 0 can be derived. Considering the sum of the diamagnetic core contributions of −37 × 10^−6^ emu mol^−1^, the Pauli paramagnetic contribution is very small. Nevertheless, well in agreement with the calculated band structure and DOS, Mg_3−*x*_Ga_1+*x*_Ir is a typical metal. The electrical resistivity *ρ*(*T*) at 300 K is ≈140 μΩ cm, while the residual resistivity of the sample is ≈35 μΩ cm. No phase transitions or superconductivity were observed down to 1.9 K.

### 3.4. Chemical Bonding

**Figure 3:**
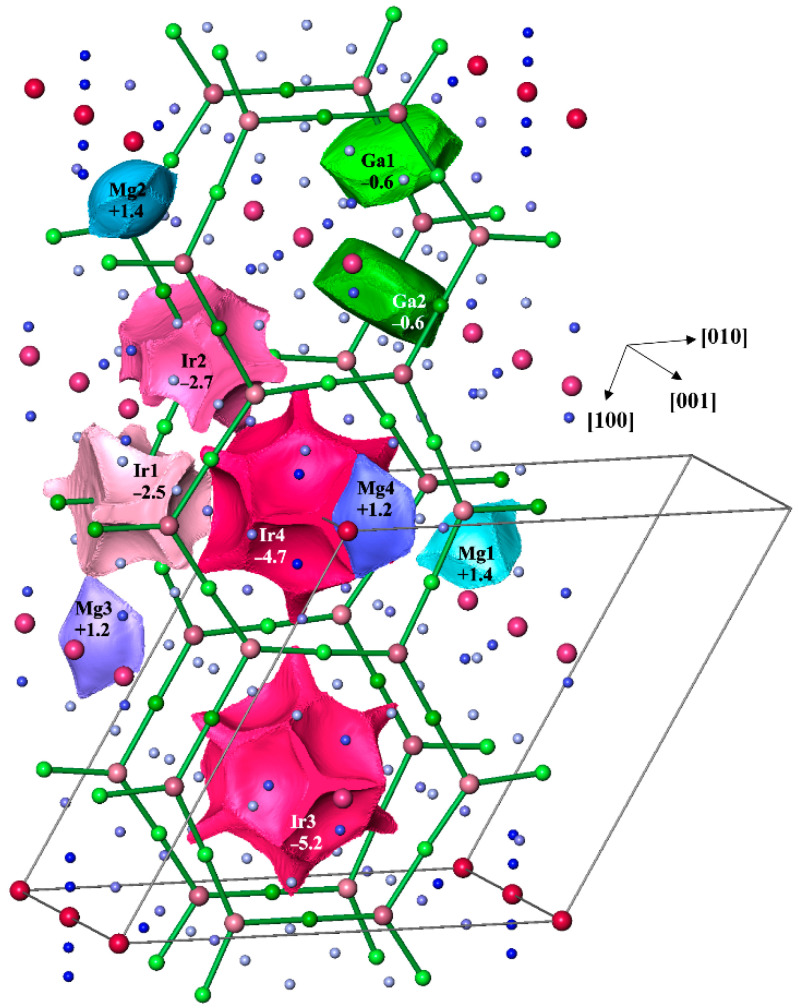
QTAIM atomic shapes and effective charges in Mg_3_GaIr. The unit cell is shown by black lines. The shortest Ga–Ir contacts (2.41–2.46 Å) are shown in green for better orientation.

Considering the low electron number in the last shell per atom (ELSA) of less than 3, which allows the assumption of the multi-atomic bonding, the analysis of chemical bonding was performed by applying quantum chemical techniques in the position space, which recently evolved to a powerful bonding investigation tool, in particular for intermetallic compounds with low VEC and multi-atomic bonding (cf. Introduction). Comparing electronegativities of the components (cf. above), one expects an essential charge transfer from Ga and Mg to Ir in Mg_3_GaIr. The effective charges of all atoms were determined from the calculated electron density. For this goal, in the position space approach, the zero–flux surfaces in the gradient vector field of the electron density form the boundaries of electron density basins, which represent atomic regions according to the Quantum Theory of Atoms in Molecules (QTAIM [29]). The shapes of the QTAIM Ir atoms in Mg_3_GaIr reveal some characteristic features (Figure 3). They are far from spherical and have concave surfaces toward neighboring magnesium species (for Ir3 and Ir4) and flatter ones toward Ga (for Ir1 and Ir2), indicating the appearance of rather different atomic interactions. The shape of the QTAIM Ga atoms is rather characteristic for covalently bonded *p*-block atoms, containing planar faces toward the neighboring iridium and slightly convex small surfaces toward the magnesium neighbors. 

The electron density was integrated in spatial regions, defined as atomic shapes in QTAIM. Their electronic populations yield the QTAIM effective atomic charges. The magnesium species have close to spherical shapes involving mostly the inner shells, and show charges of +1.2 or +1.4, playing clearly the role of cations. Following the electronegativity differences, gallium shows a relatively small negative charge (−0.6 for Ga1 and Ga2) in this structure. Iridium atoms reveal a very large negative charge if they are coordinated by Mg only (−4.7 for Ir4 and −5.2 for Ir3), and an essentially smaller one if Ga appears in their coordination sphere (−2.5 for Ir1 and −2.7 for Ir2). This variation indicates the different roles of Ir3 and Ir4 on the one hand and Ir1 and Ir2 on the other one in the structural organization of Mg_3_GaIr. 

**Figure 4:**
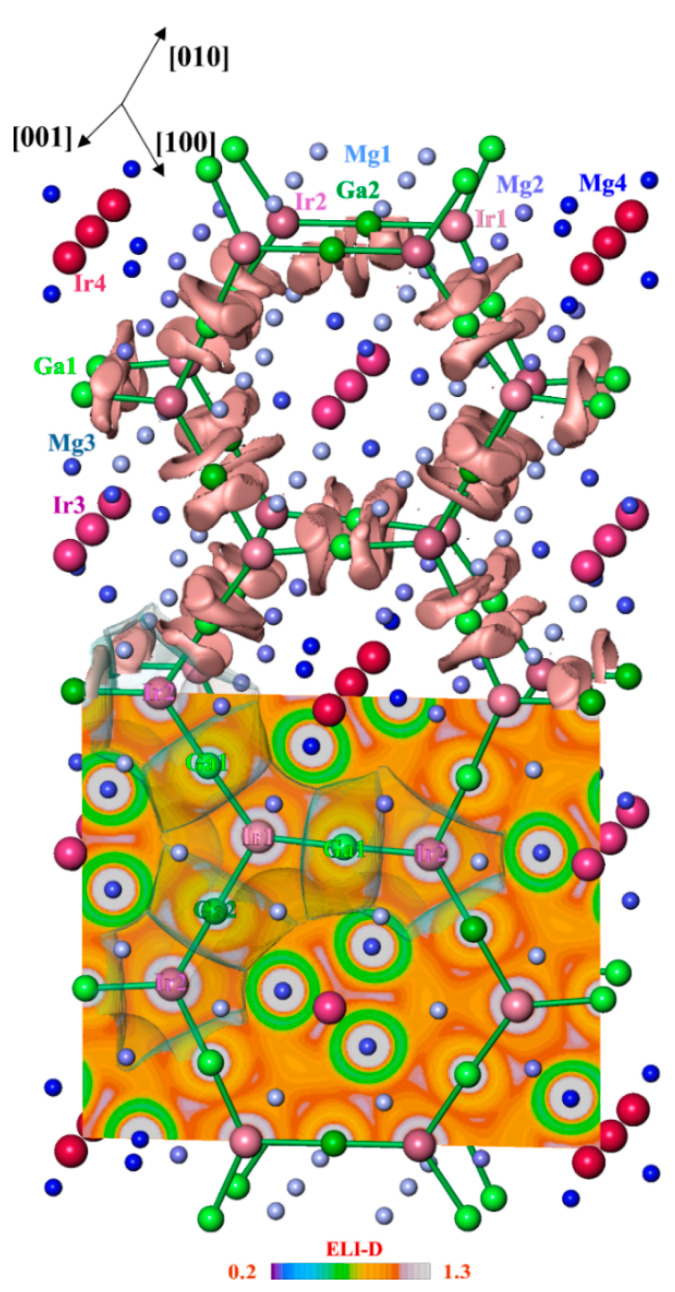
Electron localizability indicator in the anionic part of Mg_3_GaIr: (**top**) the ELI-D localization domains (isosurface) with ELI-D ≥ 1.08 visualizing the Ga–Ir bonding in the polyanion (green); (**bottom**) ELI-D distribution in the (004) plane revealing the positions of the ELI-D maxima between the Ir and Ga atoms and the intersection of ELI-D with QTAIM atomic shapes of Ir1, Ir2, Ga1 and Ga2 (grey transparent), illustrating the prevalent participation of Ir and Ga atoms in the bonding within the polyanion (two-atom bonds, left).

**Figure 5:**
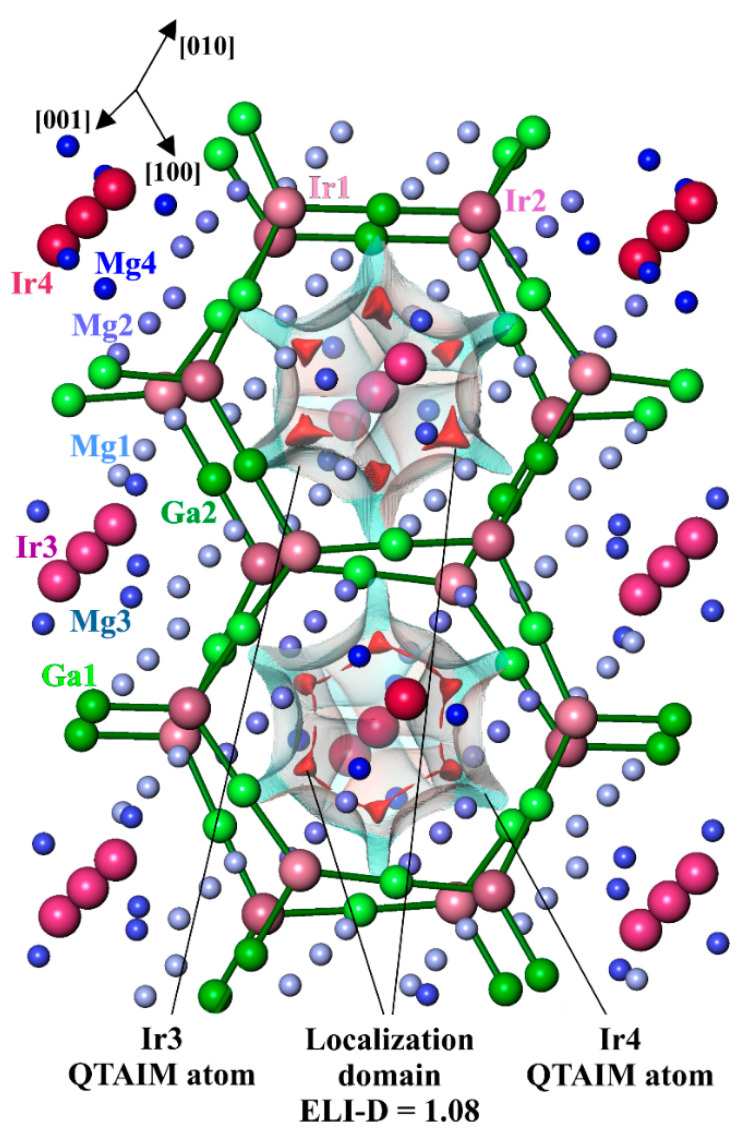
Intersection of the QTAIM atomic shapes and the localization domain with ELI-D = 1.12 in the cationic part of Mg_3_GaIr. The atomic shapes of Ir3 and Ir4 are shown in transparent grey. The isosurfaces of ELID in the vicinity of the iridium atoms (red) visualize multi-center polar Mg–Ir bonding (bonding attractors localization within the Bader atomic shapes of iridium).

Further information about the bonding between atoms was obtained by applying the electron localizability approach. The non-interacting (isolated) atoms show the spherical distribution of the electron localizability indicator (ELI-D) around the nuclei, while the local maxima (attractors) of ELI-D are located on the spheres. Regarding bonding, the spherical distribution is violated and attractors may appear in the regions of valence or penultimate shells, signaling bonding and indicating its geometrical location [23,24,26]. In Mg_3_GaIr, the ELI-D distribution in the vicinity of the atoms’ nuclei (Figure 4, lower panel) is virtually spherical in the regions of the inner shells. Several ELI-D maxima (attractors) are observed in the valence region, visualizing different components of bonding in Mg_3_GaIr. The attractors of the first group are located around the (Ga1,Ga2)–(Ir1,Ir2) contacts (the basins are shown in light pink in Figure 4, top). The populations of these bonding basins are formed mainly by the closest Ga and Ir atoms with minor contributions of the bridging magnesium species. The torus-like arrangement of the combined localization domains around the according Ga–Ir line (cf. localization domains with ELI-D ≥ 1.08 in Figure 4, top) resembles the ELI-D distribution for the two-atomic gallium transition metal bonds, e.g., in GaPd [35]. These ELI-D attractors represent heteroatomic Ga–Ir bonding within the honeycomb arrangement, which can be understood as a hierarchical derivative of the 6^3^ boron net in AlB_2_. The two-dimensional ^2^_∞_[Ga_3_Ir_2_] network contains only negatively charged atoms (cf. QTAIM analysis above), thereby representing the anionic part of the crystal structure of Mg_3_GaIr. The next group of attractors (visualized with red isosurfaces in Figure 5) represents three-atom interactions of Ir3–Mg1–Mg1, Ir3–Mg1–Mg3, Ir4–Mg2–Mg4 and Ir4–Mg2–Mg2. Thereby, Mg1 and Mg2 participate in the bonding with only one Ir atom and Mg3 and Mg4 with two Ir atoms, respectively. The basins of these attractors are to a large extent embedded into the QTAIM basins of Ir3 and Ir4, with only a small partition being in the atomic basins of the neighboring magnesium atoms. Consequently, the largest part of the population of the basins is contributed by the Ir3 and Ir4 atoms, while the magnesium contributions are rather small, indicating the strong polarity of the three-atom Ir–Mg–Mg bonds. The effective charges of the base-condensed species [(Mg1)^1.4+^]_6_[(Mg3)^1.2+^]_6/2_[(Ir3)^5.2–^] and [(Mg1)^1.2+^]_6_[(Mg3)^1.4+^]_6/2_[(Ir4)^4.7–^] are positive. Thus, this one-dimensional part ^1^_∞_[IrMg_6_Mg_6/2_] of the structure plays the role of a cation. The total composition of the unit cell for Mg_3_GaIr can be counted as [Mg_6_Mg_6/2_Ir]_6_[Ga_3_Ir_2_]_6_ = Mg_54_Ga_18_Ir_18_ = 18Mg_3_GaIr.

**Figure 6:**
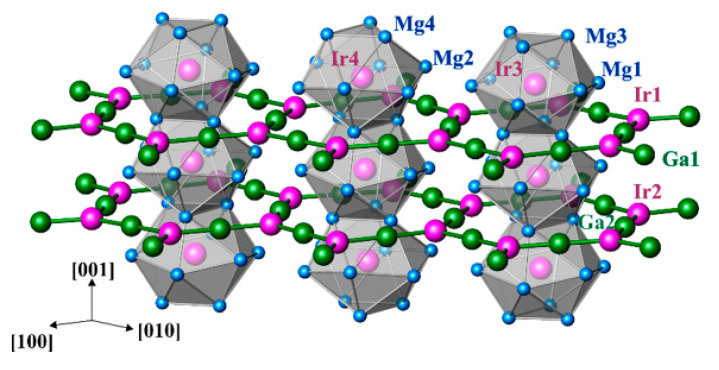
Bonding picture in Mg_3_GaIr: interplay and spatial separation of the strongly polar (ionic) bonding in the cationic substructure ^1^_∞_[Mg_6_Mg_6/2_Ir] and covalent bonding in the anionic part ^2^_∞_[Ga_3_Ir_2_]. The covalent Ga–Ir1 and Ga–Ir2 bonds are located in the planes perpendicular to [001] and shown with green lines. The honeycomb-like polyanion is interpenetrated by 1D-polycations around Ir3 (right and left) and Ir4 (middle), which run along [001] and visualized by the condensed icosahedra-bearing Ir atoms.

In summary, the analysis of chemical bonding by applying position space techniques allows one to understand the relatively complex intermetallic structure of Mg_3_GaIr as a polycationic-polyanionic architecture composed of a two-dimensional honeycomb polyanion ^2^_∞_[Ga_3_Ir_2_] running parallel to the [001] plane and a one-dimensional polycation ^1^_∞_[IrMg_6_Mg_6/2_] along [001], interpenetrating the polyanion planes (Figure 6). 

## 4. Conclusions 

In the compounds following the octet principle expressed by the equation of the generalized 8–*N* rule, polyanionic structures appear if the number of electrons in the last shell per atom is less than 4, allowing the formation of the 2e-2c bonds in the anionic substructure with characteristic examples of Zintl phases or 2e-2c and 2e-3c bonds in Lipscomb–Wade compounds. The polycationic organization is characteristic for the systems where either no covalent bonds are formed in the anionic part or the isolated anions complete a full valence shell, while the remaining available valence electrons are used for the bond formation in the cationic substructure, e.g., in (Ga_2_)^4+^(S^2–^)_2_. Recently, the formation of a polycation was found in intermetallic lutetium monogermanide LuGe, where some of the valence electrons of lutetium support the formation of the one-dimensional zig-zag anion ^1^_∞_[(2b)Ge], while the remaining ones are used for the formation of vertex-condensed polycations ^3^_∞_[Lu_4/4_]. In comparison with LuGe, Mg_3_GaIr reveals two major differences. First, it does not follow the 8–*N* rule due to the obvious lack of valence electrons. Second, both the polyanion and the polyanion are remarkably complex. Nevertheless, the general spatial organization of this intermetallic substance (e.g., the formation of polyanions and polycations) can be understood as extending principles of the 8–*N* compounds to electron-deficient compounds with multi-atomic bonding.

## Figures and Tables

**Table 1:** Crystallographic data for Mg_3−*x*_Ga_1+*x*_Ir, *x* = 0.056(7).

Composition	Mg_2.944_Ga_1.056_Ir
Space group	*P*6_3_*/mcm* (no. 193)
Pearson symbol	*hP*90
Formula units per unit cell, *Z*	18
Lattice parameters ^a^	
*a*/Å	14.4970 (3)
*c*/Å	8.8638 (3)
*V*/Å^3^	1613.3 (1)
Calc. density/g cm^−1^	9.00
Crystal shape	prism-like
Crystal size/mm^3^	0.030 × 0.030 × 0.125
Diffraction system	RIGAKU AFC7
Detector	Mercury CCD
Radiation, *λ*/Å	Mo*Kα*, 0.71073
Scan; step/degree; *N* (images)	*φ*, *ω*, 0.6; 500
Maximal 2*θ*/degree	63.0
Range in *h*, *k*, *l*	−21 ≤ *h* ≤ 16
	−20 ≤ *k* ≤ 20
	−12 ≤ *l* ≤ 12
Absorption correction	multi-scan
*T* (max)/*T* (min)	3.60
Absorption coefficient/mm^−1^	47.9
*N* (*hkl*) measured	13000
*N* (*hkl*) unique	961
*R* (int),	0.061
*N* (*hkl*) observed	918
Observation criteria	*F* (*hkl*) > 4*σ* (*F*)
Refined parameters	49
*R* (*F*) GOF	0.027 1.04
Residual peaks/e Å^−3^	−1.90/1.45

^a^ Guinier powder data (Co*K*α1 radiation, internal standard LaB_6_, *a* = 4.15692 Å).

**Table 2:** Atomic coordinates and equivalent displacement parameters (in Å^2^) in the crystal structure of Mg_3−*x*_Ga_1+*x*_Ir.

Atom	Site	*x*	*y*	*z*	*U*_eq_
Mg1	24*l*	0.1245 (2)	0.4566 (2)	0.0241 (3)	0.0152 (8)
Mg2	12*k*	0.2101 (3)	0	0.5261 (4)	0.0151 (11)
Mg3 *	12*j*	0.3391 (3)	0.5577 (3)	¼	0.0122 (10)
Mg4 *	6*g*	0.1118 (3)	0	¼	0.0139 (13)
Ir1	6*g*	0.31683 (4)	0	¼	0.0082 (2)
Ir2	6*g*	0.65164 (4)	0	¼	0.0091 (2)
Ir3	4*d*	⅓	⅔	0	0.0098 (2)
Ir4	2*b*	0	0	0	0.0109 (2)
Ga1	12*j*	0.17485 (9)	0.33560 (9)	¼	0.0127 (4)
Ga2	6*g*	0.4863 (1)	0	¼	0.0112 (4)

* Occupancies: 0.951(6) Mg + 0.049 Ga for Mg3 and 0.929(9) Mg + 0.071 Ga for Mg4, respectively.

**Table 3:** Selected interatomic distances (in Å) in Mg_3−*x*_Ga_1+*x*_Ir.

Atoms		*d*, Å	Atoms		*d*, Å
Mg1 –	1Ir1	2.778 (3)	Mg4 –	2Ir4	2.746 (1)
	1Mg3	2.812 (3)		2Mg4	2.808 (3)
	1Ga2	2.869 (3)		2Mg2	2.831 (3)
	1Ir2	2.965 (2)		2Ga1	2.954 (2)
	1Ga1	2.983 (3)		1Ir1	2.972 (2)
	1Mg1	3.039 (4)		4Mg2	3.302 (3)
	1Ir3	3.043 (3)			
	1Mg1	3.092 (4)	Ir1 –	2Ga1	2.410 (1)
	1Mg1	3.127 (4)		1Ga2	2.456 (1)
	1Mg2	3.127 (4)		4Mg1	2.778 (3)
	1Ga1	3.190 (3)		2Mg2	2.895 (3)
	1Mg3	3.256 (4)		1Mg4	2.972 (2)
	1Mg3	3.358 (4)			
	1Ga2	3.368 (3)	Ir2 –	1Ga2	2.398 (1)
				2Ga1	2.428 (1)
Mg2 –	1Ir2	2.821 (3)		2Mg2	2.821 (3)
	1Mg4	2.831 (3)		4Mg1	2.965 (3)
	1Ir1	2.895 (3)		2Mg3	3.104 (4)
	2Ga2	2.905 (3)			
	1Ir1	3.054 (2)	Ir3 –	6Mg3	2.747 (2)
	2Mg2	3.080 (3)		6Mg1	3.043 (3)
	2Mg1	3.127 (3)			
	2Mg4	3.302 (3)	Ir4 –	6Mg4	2.746 (1)
	2Ga1	3.333 (3)		6Mg2	3.054 (2)
					
Mg3 –	2Ir3	2.747 (2)	Ga1 –	1Ir1	2.410 (1)
	2Mg3	2.812 (5)		1Ir2	2.428 (1)
	2Mg1	2.812 (3)		1Mg3	2.893 (4)
	1Ga1	2.893 (4)		2Mg2	2.905 (3)
	1Ga2	2.903 (4)		1Mg4	2.954 (2)
	1Ir2	3.104 (4)		2Mg1	2.983 (3)
	2Mg1	3.256 (4)		2Mg1	3.190 (3)
	2Mg1	3.358 (4)		2Mg2	3.333 (3)
					
			Ga2 –	1Ir2	2.398 (1)
				1Ir1	2.456 (1)
				4Mg1	2.869 (3)
				2Mg3	2.903 (4)
				4Mg1	3.368 (3)

## Data Availability

The crystallographic data are available at the CSD Data Bank under the reference number 2129909.

## Supplementary Materials

The following are available online, Figure S1: Temperature dependent magnetic susceptibility of Mg_3−*x*_Ga_1+*x*_Ir annealed at 500 °C and 600 °C.

## Sample Availability

Samples of the compounds are not available from the authors.
