# Supplementary material for: Polycation–Polyanion Architecture of the Intermetallic Compound Mg3−xGa1+xIr
Source: Molecules. 2022 Jan 20;27(3):659. doi: 10.3390/molecules27030659 (PMC8840708; doi:10.3390/molecules27030659)
Supplement: Supplementary file 1 [file molecules-27-00659-s001.zip › molecules-1543267-supplementary.pdf]

Supporting information

Polycation–Polyanion Architecture of the Intermetallic Compound $Mg_{3-x}Ga_{1+x}Ir$

Olga Sichevych, Yurii Prots, Walter Schnelle, Frank R. Wagner and Yuri Grin *

Max-Planck-Institut für Chemische Physik fester Stoffe, Nöthnitzer Str. 40, 01187 Dresden, Germany; olga.sichevych@cpfs.mpg.de (O.S.); prots@cpfs.mpg.de (Y.P.); schnelle@cpfs.mpg.de (W.S.); Frank.Wagner@cpfs.mpg.de (F.R.W.)
* Correspondence: grin@cpfs.mpg.de; Fax: +49-351-46464002

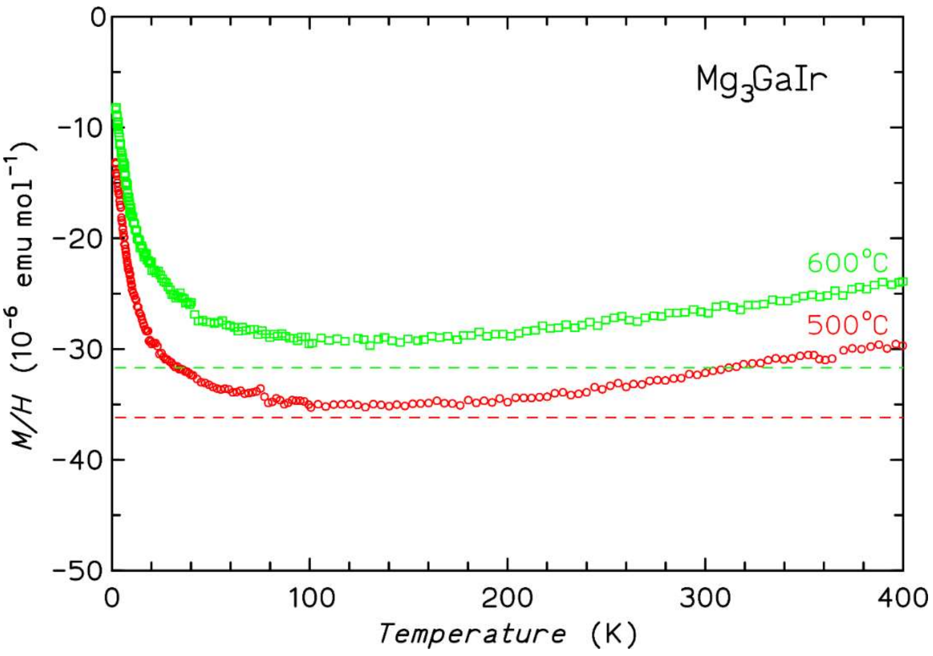

Figure S1. Temperature dependent magnetic susceptibility of $Mg_{3-x}Ga_{1+x}Ir$ annealed at 500 °C and 600 °C.
